# Dr. William H. Byford

**Published:** 1890-07

**Authors:** 


Dr. William H. Byford, of Chicago, died in that city May 20, 1890, aged 73 years, after an attack of augina pictoris, lasting but two hours. Dr. Byford has been a conspicuous figure in the medical history of his period. Distinguished as an author and a teacher he leaves a name and a fame that will outlast the centuries.

